# When populism takes over the delivery of health care: Venezuela

**DOI:** 10.3332/ecancer.2017.ed73

**Published:** 2017-11-23

**Authors:** Sunil Daryanani

**Affiliations:** Yeovil General Hospital NHS Foundation Trust, Somerset BA21 4AT, UK and; Hospital de Clínicas Caracas, Consultorio 304, Avenida Panteón, San Bernardino, Caracas 1011, Venezuela

**Keywords:** health care, Venezuela, populism, cancer

## Abstract

Adequate and modern health care is not available in Venezuela at this moment. A humanitarian crisis of post war dimensions is currently rampant and afflicting all Venezuelans alike. The delivery and availability of cancer care is severely limited and inadequate. No funding is available as populist measures, mismanagement, pillaging, corruption, lack of forethought and expertise have brought the country into severe economic collapse and political turmoil.

## Background and political scenario

Adequate and modern health care delivery at this moment is not existent or available in Venezuela. Despite recent coverage in both the medical press and the lay press of an ongoing crisis extending for many months with riots and general unease, with more than a hundred and fifty deaths arising from these protests due to public inconformity and opposition to unconstitutional government measures of late, this is not a new problem or crisis. Venezuelan medicine and health care have been afflicted by a scourge of problems for more than two decades. A humanitarian crisis of post war dimensions is currently rampant and afflicting all Venezuelans alike [1]. Sadly, other countries in the world are going through similar barbaric situations, such as Syria [2], and the world remains expectant and paralysed.

Being both Venezuelan born as well as educated and trained in the Venezuelan system more than thirty years ago, the country (at that time) seemed destined to forge a great infrastructure manned by a wealth of highly trained and committed individuals. In a country where the highest oil reserves are concentrated under slightly less than a million square kilometres all seemed possible. A rising oil price, a privileged geographical position, the longest running democracy in the region, perfect weather with natural beauty and mostly free high quality university education as well as opportunities and scholarships to train abroad at centres of excellence: this was a country with no parallel. Postgraduate students never harboured the slightest intent of remaining abroad but always planned to return to Venezuela and be part of its proud transformation.

Certain social inequalities were evident. A significant segment of the population lacked access to health care, education and housing. Generations of politicians delivered empty promises, certain social programmes were instituted, but alas, not available to all and with limited cover.

Despite this rather incongruous backdrop the general scenario was one of buoyancy, wealth and wellbeing. Venezuela became the ‘jewel in the crown’ for the whole sub region (from Mexico to Argentina) and an enormous influx of immigrants made this their new home, attracted by its wealth and work opportunities. A country of multiple contrasts, it showed elements of the First World interspersed with elements of a Third World country all within a few metres of each other.

Right in the middle of this cultural and historical mélange emerged the figure of Hugo Chávez (1954-2013) who with charisma and the backing of a forgotten few won the favour of the general masses of the Venezuelan underprivileged. Blinded by his rather unconventional approach to politics, a full purse (oil prices at a maximum historic level), an array of allies (with no other work or moral ethics than easy money) began changing the landscape dramatically by the introduction of social reforms similar to Brazil (driven by the now disgraced Lula da Silva) won the favour and admiration of the western world. Unfortunately the world at large turned its back on unconventional economic measures, takeovers, repeated disrespect to the Constitution and prevalent laws, growing violence and the discontent of half the country. Behind this, and in a rather surreptitious role, was Cuba who with the great admiration proffered by Chavez offered help and assistance in exchange for free oil and other privileges.

## Health care in Venezuela

In the last two decades health care in Venezuela has dwindled even further despite its enormous, albeit dwindling, economical bonanza. Over fifteen health ministers have been named to head the Ministry of Health in these almost two decades. Statistics and health indicators have all been misused, falsified or published (or not published at all with a data embargo) using misconstrued data to the point that the Food and Agriculture Organization of the United Nations awarded a prize to Venezuela over two years ago on feeding initiatives when famine was already evident [3–4].

Recently a now dismissed Health Minister known for her allegiance to the political regime authorised the publication of a much delayed Epidemiological Bulletin that confirmed that conditions such as malaria [5], diphtheria and tuberculosis, among others, were rampant. Furthermore, indicators pertaining to child and maternal mortality (grossly under-reported as all official figures are) had greatly increased; this report created alarm worldwide [6].

Health care in Venezuela is split into three systems: a public system open to all comers, a National Insurance system (named Instituto Venezolano de los Seguros Sociales, IVSS) and a private health system that comprises both small and large hospitals and private offices. Over 50 years ago patients favoured undergoing complex and delicate procedures in both public and IVSS hospitals and attended clinics and outpatients in private medicine. Over the years, with further and greater investment in the private sector this managed to outrank the other two public options.

As Chavez began to rule he corralled what would be his new ‘revolutionary’ health care system with a parallel primary care system (the ‘Barrio Adentro’ programme which translated would imply ‘Deep inside the slums’) and new universities churning out doctors after three years of training against the established system with primary care running all the way to highly specialised care. Public and IVSS hospitals in the process began to suffer from budget shortages and cuts as well as rampant corruption and the baton of health was taken over by the private health care system. At a certain moment, Chavez openly attacked the private health care system but as he himself noted, his communist/socialist adepts preferred receiving health care in private hospitals than in public state hospitals. Chávez himself did not resort to his own medical system when diagnosed with cancer and chose to be treated in Cuba under very mysterious and undisclosed circumstances.

The Chavismo (i.e. Chavez´s movement) depended outright on private medicine and despite overt threats of appropriation and hostile takeovers, a silent stalemate was reached.

For over fifteen years foreign exchange has been under the tight control of the central government. No entity, either private or corporate, is allowed any foreign funds under the official exchange rate unless they are chosen as sympathisers or allies of the government. This has forced the development of a black market to obtain foreign currency. Venezuela relies basically on imports as befits most oil exporting countries and its internal industrial apparatus has been chronically stripped and dismantled by the Chavismo and its erratic economic policies.

Venezuelan nationals for many years were allowed up a maximum of USD 2,500 for travel per year and USD 150 for use on internet purchases. Any further acquisition of foreign currency by any other means is considered illegal and therefore unlawful. Currently, and for the last three years, the paperwork and the possibility of obtaining funds is virtually inexistent making foreign travel almost impossible.

Central to this situation, from a medical standpoint, is health care provision and food availability. Patients are dying from common illnesses. Patients with complex conditions are not able to obtain adequate care. Hospitals are bare. Stocks of medical supplies are down to a minimum. In the largest University hospital in the country, vendors in the corridors sell medical supplies as hospitals are powerless to offer basic supplies. This is the sort of situation which no one in the most rural and deprived settings in the western world is likely to face.

Notwithstanding this, the general public is exposed to rampant crime and violence which has made Venezuela one of the most violent places on Earth, and furthermore impunity is the rule with less than 5% of violent crimes investigated. Recently, the National Academies were ransacked and pillaged by government paramilitary hordes with no other intention but to demonstrate contempt and disrespect to the academic establishment [7]. Inflation is the highest in the world, and despite government widespread censorship, it is over 700% annually. Currently, the minimum wage is USD 12 a month. The country is in a desperate situation.

Hunger is the norm with several serious studies reporting at least an eight kilogramme loss among adults [8]. Long queues are seen on a daily basis as some basic foodstuffs are available at the official rate and other articles are imported at the unofficial rate which is more than 80 times over that price [9]. Disparities are the norm. It is a way to subjugate the general public.

Medicines are very difficult to obtain. Essential antibiotics, contraceptive pills, analgesics, and drugs for high blood pressure are not available. Major pharmaceutical firms have had to adapt from Venezuela being the most important market in Latin America to just having a minimal presence and no staff.

## Cancer care in Venezuela

In a populist move, Chávez promoted and revamped a programme of free cancer drugs to all Venezuelans and residents (Medicinas de Alto Costo, High-cost medicines) which was the envy of much of the continent. Monoclonal antibodies, tyrosine kinase inhibitors, anti-HIV drugs and all new and expensive drugs were dished out without much more than a handwritten medical prescription. Public cancer hospitals did not (and still do not) have the possibility of carrying out basic immunohistochemistry stains on tissue samples or proper imaging (CT or MRI scans) but patients were getting state-of-the-art chemotherapy regimens for advanced colorectal cancer with no possibility of appraisal of response or surgery for metastatic disease. More often than not monoclonal antibodies were available but simple drugs such as 5-Fluorouracil or Leucovorin, or basic drugs such as platinum salts or doxorubicin, were scarce or unavailable, rendering treatment regimes incomplete or impossible to administer. Patients with breast cancer could opt for trastuzumab without knowledge of HER2 status.

Pharmaceutical companies sold all their imports to the government and neglected their other marketing allies. The state became such an overarching giant that marketing strategies had no other option than to place products directly with the government. Payment was delayed and currently companies have had to absorb multimillion debts and stop imports.

Cancer patients are more often than not having to stop treatment or buy or import their medications at unmanageable costs to them [10]. Patients are resorting to alternative or natural remedies or to religion in some cases for comfort as treatments, medical supplies and procedures are not available. Despite the evidence, the government and its officials continue to advertise and report that all is normal and blatantly deny any ongoing crises.

Cancer care in Venezuela is severely disjointed from prevention to diagnosis to treatment and to end-of-life care to a degree incompatible with dignity as we know it. A humanitarian crisis is being ignored and basic human rights are being violated.

## Conclusion

Even in the best of circumstances, should there be a turn to a democratic based government, it will be a few years until Venezuelans are offered an adequate medical system with a reasonable standard of health care. This is the sort of social experiment that should not have happened. Many lessons could be learnt from this and no other country or society in the world should ever have to go through anything like this.

The access to proper medical care is a right of all. Health care systems should be solid and not submitted to frailty and mismanagement. The desperate plight of the Venezuelans needs to be publicised and reported as a politically induced crisis and one that could have been averted if a solid health care system with its overseers had been in place. The death toll of the current humanitarian crisis is enormous and cancer patients are some of its major victims.
